# Predictors of Weekday and Weekend Screen Time in a Clinical Child and Adolescent Psychiatric Sample (Aged 12–18 Years)

**DOI:** 10.3390/children13070875

**Published:** 2026-06-30

**Authors:** Helena Gampe, Lucas Rainer, Belinda Plattner, Kornelius Winds

**Affiliations:** 1 Department of Child and Adolescent Psychiatry and Psychotherapeutic Medicine, University Clinics Salzburg, Paracelsus Medical University Salzburg, Ignaz-Harrer-Straße 79, 5020 Salzburg, Austria; helena.gampe@stud.plus.ac.at (H.G.); lucas.rainer@teach.pmu.ac.at (L.R.); belinda.plattner@teach.pmu.ac.at (B.P.); 2 Department of Psychology, University of Salzburg, Hellbrunner Straße 34, 5020 Salzburg, Austria; 3 Centre for Cognitive Neuroscience Salzburg, University of Salzburg, Ignaz-Harrer-Straße 79, 5020 Salzburg, Austria; 4 Department of Neurology, Neurocritical Care and Neurorehabilitation, University Clinics Salzburg, Member of the European Reference Network EpiCARE, Paracelsus Medical University Salzburg, Ignaz-Harrer-Straße 79, 5020 Salzburg, Austria

**Keywords:** screen time, problematic use of the internet, externalizing symptoms, fear of missing out, child and adolescent psychiatry, clinical sample, emotion regulation

## Abstract

**Highlights:**

**What are the main findings?**

- Problematic use of the internet (PUI) was the strongest and most consistent predictor of both weekday and weekend screen time in a clinical child and adolescent psychiatric sample. PUI alone accounted for 15.0% of the variance in weekday screen time.
- Externalizing symptoms predicted weekday but not weekend screen time. This pattern may reflect context-specific differences and could be related to the daily structure imposed by school obligations.

**What are the implications of the main findings?**

- Clinical assessment of digital engagement in adolescents should prioritize PUI over aggregate screen time duration, as screen time alone fails to capture the compulsive quality of digital engagement in this population.
- Screen time reduction interventions are unlikely to be sufficient without addressing the underlying motivational and regulatory mechanisms driving excessive digital engagement, particularly PUI, in clinically referred youth.
- Structured daily routines in inpatient and day clinic settings may serve a protective function by limiting the expression of compulsive or symptom-driven digital engagement, a pattern that, if replicated, may have relevance for therapeutic program design.

**Abstract:**

**Background/Objectives**: Screen time in children and adolescents has become a prominent public health concern, yet most research has focused on community samples, leaving clinically referred youth underrepresented. This study examined predictors of weekday (WD-ST) and weekend screen time (WE-ST) in a clinical child and adolescent psychiatric sample, with a particular focus on problematic use of the internet (PUI), externalizing symptoms, and fear of missing out (FoMO). **Methods**: A retrospective secondary analysis of pooled datasets from multiple clinical studies was conducted with 173 adolescents (66.5% female; age range 12–18 years) receiving child and adolescent psychiatric treatment at the University Hospital of Salzburg, Austria. Multivariate linear regression analyses examined self-esteem, adaptive and maladaptive emotion regulation strategies, internalizing and externalizing symptoms, FoMO, and PUI as predictors of WD-ST and WE-ST separately. *p*-values were adjusted for multiple comparisons using the Benjamini–Hochberg False Discovery Rate correction. **Results**: In the follow-up hierarchical regression models, PUI was the strongest and most consistent predictor across both models, independently explaining 15.0% of variance in WD-ST time and remaining the only significant predictor in the final WE-ST. Externalizing symptoms significantly predicted WD-ST (β = 0.219, *p* = 0.021) but not WE-ST. FoMO showed a consistent positive association with WD-ST across both regression models, though this did not reach statistical significance. Self-esteem, emotion regulation strategies, and internalizing symptoms were not significantly associated with screen time in either model. **Conclusions**: Screen time in clinical adolescent populations cannot be adequately captured by duration alone. PUI reflects a compulsive quality of digital engagement independent of broader psychopathological burden, and the observed difference in weekday versus weekend predictors is consistent with a potential role of daily structure, though this was not formally tested in the present study. Routine clinical assessment should prioritize PUI-focused evaluation over aggregate screen time as a more sensitive indicator of clinically relevant digital engagement.

## 1. Introduction

Discussions about restricting social media access for specific age groups have become increasingly prominent in political and public discourse. This issue is often framed in terms of reducing screen time as a strategy to protect child and adolescent mental health. Across the European Union, there is a growing trend towards political and regulatory measures aimed at restricting or more strongly regulating children’s and adolescents’ access to social media platforms. Current initiatives focus particularly on age verification systems, minimum-age requirements, and the regulation of potentially addictive platform features within the framework of the Digital Services Act [1]. Together, these developments reflect a growing political consensus that reducing screen exposure is a central pathway to improving youth well-being. However, the empirical evidence suggests a considerably more nuanced relationship between screen use, digital environments, and mental health outcomes.

While excessive digital media exposure has been associated with a range of adverse health outcomes across multiple domains [2], the association between digital technology use and adolescent well-being appears negative but small in magnitude, explaining at most 0.4% of the variation in well-being [3]. Moreover, the evidence base underpinning blanket restriction policies remains inconclusive, with most studies unable to establish causality, suggesting that prohibition alone is unlikely to be sufficient [4]. However, these policy debates are informed primarily by community and school-based research, and it remains unclear whether similar patterns extend to clinically referred youth with greater psychopathological burden and more complex digital engagement.

Internet use has become an integral part of the daily lives of children and adolescents, and its potential harms are increasingly documented across community samples. Clinically referred adolescents, however, represent a population in which these dynamics are likely amplified, as higher rates of emotional dysregulation, maladaptive coping, and clinical burden create conditions in which digital engagement may assume qualitatively different functions than in the general population [5,6]. In Europe approximately 92% of young people aged ten years and older own a smartphone [7], raising concerns about the effects of digital media use on the development of children and adolescents [8]. Nearly half of adolescents in high-income countries report near-continuous online presence [9].

Problematic use of the internet (PUI), a term used interchangeably with “problematic usage of the internet” and related constructs across the literature [10], refers to maladaptive patterns of internet engagement characterized by impaired control and functional impairment in social, academic, or everyday domains [11]. The World Health Organization (WHO) Regional Office for Europe, drawing on data from the Health Behaviour in School-aged Children (HBSC) study involving 279,117 adolescents across 44 countries, reported a rise in problematic social media use from 7% in 2017/2018 to 11% in 2021/2022, with 13% of girls and 9% of boys classified as problematic users [12]. These figures represent community samples and are likely to underestimate the burden in clinical settings, where prevalence rates are considerably higher. Studies in adolescent psychiatric inpatient populations report rates of subthreshold PUI exceeding 60% and full PUI of approximately 29–35% [5,6], identifying a population whose digital engagement patterns warrant specific clinical attention.

Although screen time and PUI are related constructs, they represent distinct constructs. Screen time refers to the amount of time spent using digital devices [13], whereas PUI captures maladaptive patterns of engagement characterized by impaired control and functional impairment [11]. Associations between screen time and health outcomes appear to depend more on the type, content, and context of use than duration alone [14]. Longitudinal evidence reports generally modest associations between screen time and internalizing symptoms, with stronger effects observed for interactive technologies such as smartphones and computer-based internet use than for passive media use [15].

Consistent with the Interaction of Person–Affect–Cognition–Execution (I-PACE) model, PUI is conceptualized as emerging from the interaction of individual vulnerability factors, including psychopathological burden, personality traits, and maladaptive coping and affective-cognitive responses to specific online stimuli [16,17]. Compensatory internet use to regulate negative emotional states has been proposed as a central mechanism across all stages of this process.

Gender shapes these patterns in important ways. Although overall PUI severity may not differ significantly between boys and girls, the type and motivational context of internet use vary, with boys predominantly using the internet for gaming, while girls prioritize social communication and social media engagement [18,19]. Studies relying on generalized PUI measures may underestimate gender-specific differences, as associations related to internet gaming disorder in boys and social media addiction in girls may be obscured when data are aggregated across genders [19]. Beyond individual factors, the family context is an important determinant of adolescent PUI. Parental PUI is among the strongest predictors of adolescent PUI, and evidence suggests that the quality of parent–child communication regarding internet use may constitute a stronger protective factor than screen time restrictions alone [20].

Alongside gender and family associated factors, psychosocial aspects have emerged as important contributors to the development of PUI. Empirical findings indicate that PUI is associated with both internalizing and externalizing behavior problems [21]. Longitudinal evidence further suggests that externalizing symptoms predict subsequent increases in screen time, particularly on weekdays, in community-based adolescent samples [22]. Moreover, associations between screen time and behavioral outcomes vary depending on study design and the operationalization of screen-related constructs [14]. At the more severe end of the clinical spectrum, problematic digital media use has been associated with self-harm and suicidality in adolescents. An integrative review concluded that excessive screen exposure during the COVID-19 pandemic was consistently associated with increased self-harm, suicidal ideation, and suicidal behavior, particularly in the context of intensive smartphone and social media use [23]. These associations are likely more pronounced in clinical samples with higher baseline psychopathological burden [5,6].

The role of self-esteem in PUI remains poorly understood. Self-esteem, defined as the subjective evaluation of one’s own worth as a person [24], is associated with positive developmental outcomes including healthy relationships, adaptive coping and occupational success [25,26]. Adolescence represents a particularly vulnerable period for self-esteem development due to heightened social comparison, peer evaluation, and ongoing maturation of self-regulatory capacities [27,28]. Emotional regulation difficulties are especially prevalent during this developmental stage, as the prefrontal cortical systems underlying impulse control and affect regulation are not yet fully developed, leaving adolescents more susceptible to externally driven emotional compensation strategies [29]. These characteristics may render adolescents particularly vulnerable to maladaptive patterns of digital engagement, as online environments offer accessible avenues for social belonging and emotional compensation.

Studies examining the relationship between screen-based device use and self-esteem report negative associations, particularly for social media use, although these associations appear to be moderated by platform, usage type, and individual characteristics [30,31,32]. In clinical samples, where internalizing symptoms and social difficulties are elevated, low self-esteem may function both as a vulnerability factor for and a consequence of depressive symptoms and PUI [33], highlighting its potential relevance in this clinical population.

Beyond self-esteem, fear of missing out (FoMO) has emerged as another psychological factor associated with PUI. Defined as the apprehension that others may be having rewarding experiences from which one is absent, FoMO has been identified as a motivational driver of social media engagement and internet use [34]. Adolescents may be particularly vulnerable given the developmental importance of peer belonging, social comparison and sensitivity to social feedback [35,36]. Given the centrality of peer relationships during adolescence, digital environments may assume particular importance as contexts for maintaining social connectedness and monitoring peer interactions. Consequently, FoMO may represent an important mechanism linking social concerns to increased digital engagement and screen use during adolescence. Examining FoMO may be particularly relevant in a clinical adolescent sample, where social and emotional difficulties may increase vulnerability to FoMO-related online behaviors.

Despite growing evidence regarding PUI and screen-related behavior in community samples, comparatively little is known about factors associated with screen time in clinically referred adolescents. The present study therefore examines predictors of weekday (WD-ST) and weekend screen time (WE-ST) in a clinical child and adolescent psychiatric (CAP) sample, focusing on PUI, externalizing symptoms, and FoMO. Based on the I-PACE model and prior empirical evidence, we hypothesized that PUI would emerge as the strongest predictor of both WD-ST and WE-ST, reflecting its role as an index of compulsive digital engagement driven by impaired self-regulation [16,17]. We further hypothesized that externalizing symptoms would be positively associated with screen time, particularly on weekdays, given that school-imposed daily structure may constrain but not eliminate symptom-driven digital engagement [21]. FoMO was expected to show a positive association with screen time, consistent with its role as a need-based motivator of social media engagement in adolescents [34].

## 2. Materials and Methods

The present study employed a retrospective secondary analysis of pooled datasets to examine the association between intrapersonal factors (e.g., self-esteem), psychosocial factors (e.g., adaptive and maladaptive emotion regulation strategies, internalizing and externalizing symptoms), FoMO, PUI, and WD-ST as well as WE-ST. For this purpose, datasets from several previously conducted studies were combined and synthesized into one common dataset. All included studies were approved by the respective local ethics committees and conducted in accordance with the Declaration of Helsinki. Prior to data integration, datasets were checked for duplicate participants to ensure that each participant was included only once in the final analytic sample.

### 2.1. Procedure of Data Synthesis

Data from four earlier research projects conducted at the CAP Department of the University Clinics Salzburg, Paracelsus Medical University, Austria, were merged. Participants were recruited from inpatient, day-clinic treatment and out-patient settings. In the inpatient setting, the clinic follows a systemic participatory multiprofessional treatment approach with structured daily routines, including individual and group-based therapeutic interventions, school attendance and guided leisure time activities. Once sufficient clinical stability is achieved, most patients are permitted to spend weekends at home in order to apply and evaluate strategies acquired during treatment in their everyday environment. Patients treated at the clinic ranged in age from 6 to 18 years; however, for the studies included in the present analysis, only adolescents aged 12–18 years were eligible for participation. The following studies were included:

1. “Effects of dance art on self-esteem and body perception in children and adolescents: A pilot study” (Ethics Committee of Salzburg, No. 1054/2023),
2. “Me and myself–Self-esteem, self-image, and portrait photography in children and adolescents: A pilot study” (Ethics Committee of Salzburg, No. 1091/2021),
3. “Psychological and physiological effects of choir singing on mentally ill, socially underprivileged, and privileged healthy children and adolescents: An open-label, single-arm controlled study” (Ethics Committee of Salzburg, No. 2554/2-2019),
4. “Retrospective quality evaluation of an outpatient DBT skills group” (Ethics Committee of Paracelsus Medical University, No. 2024-0020).

All contributing studies were conducted at the same child and adolescent psychiatric service and used comparable recruitment procedures. The dataset from study 4 served as the primary dataset to which the remaining datasets were sequentially merged. Characteristics of the contributing studies and the number of complete cases available for the regression analyses are presented in Table 1. Studies were included if they contained measures relevant to the constructs examined in the present investigation. Following data harmonization, listwise deletion was applied for the regression analyses. Because the full and follow-up models included different sets of predictors, the number of complete cases varied across analyses depending on variable availability across the contributing studies.

To ensure data integrity, datasets were screened for duplicate participants based on matching birth dates and sex. Given that all datasets originated from the same institution, the likelihood of undetected duplicates was considered low. Duplicate entries identified through this procedure were removed to ensure that each participant was included only once in the final analytic dataset. Participants younger than 12 years or older than 18 years were excluded from the sample (N = 5).

### 2.2. Instruments

#### 2.2.1. Self-Esteem Inventory for Children and Adolescents (German: Selbstwertinventar für Kinder und Jugendliche) (SEKJ)

Self-esteem was measured using the self-worth level subscale (German: Selbstwerthöhe) of the Self-esteem Inventory for Children and Adolescents (German: Selbstwertinventar für Kinder und Jugendliche) (SEKJ) [37], a standardized self-report instrument assessing different facets of self-esteem. Items are answered on a 5-point Likert scale, with higher values indicating higher self-esteem. Previous studies reported good internal consistency for the SEKJ scales, with Cronbach’s α ranging between 0.81 and 0.90.

#### 2.2.2. Youth Self-Report (YSR/11-18R)

Internalizing and externalizing symptoms were assessed using the German version of the Youth Self-Report (YSR/11–18R) [38], a standardized self-report questionnaire measuring emotional and behavioral problems in adolescents. Internalizing symptoms encompass emotional difficulties such as anxiety and depressive symptoms, whereas externalizing symptoms comprise behavioral problems such as aggressive and rule-breaking behavior. The instrument comprises syndrome scales as well as broadband scales for internalizing and externalizing problems. Items are rated on a 3-point scale ranging from 0 (not true) to 2 (very true or often true). Previous studies demonstrated acceptable to good internal consistencies for the YSR scales, with Cronbach’s α values ranging from 0.71 to 0.88.

#### 2.2.3. Questionnaire for the Evaluation of Emotional Regulation in Children and Adolescents (German: Fragebogen zur Erhebung der Emotionsregulation bei Kindern und Jugendlichen) (FEEL-KJ)

Emotion regulation strategies were assessed using the Questionnaire for the Evaluation of Emotional Regulation in Children and Adolescents (German: Fragebogen zur Erhebung der Emotionsregulation bei Kindern und Jugendlichen) (FEEL-KJ) [39,40], a standardized self-report questionnaire measuring adaptive and maladaptive emotion regulation strategies for the emotions anger, anxiety, and sadness. Items are rated on a 5-point Likert scale ranging from almost never to almost always. In the present study, the adaptive and maladaptive secondary scales were used. Previous studies reported good internal consistencies for the FEEL-KJ scales, with Cronbach’s α values ranging from 0.69 to 0.91.

#### 2.2.4. Fear of Missing out Scale

FoMO was assessed using the Fear of Missing Out Scale [34], a 10-item self-report questionnaire measuring the extent to which individuals experience concerns about missing rewarding social experiences and the desire to remain continuously connected with others. Items are rated on a 5-point Likert scale, with higher scores indicating higher levels of FoMO. The scale has demonstrated good internal consistency and reliability in previous studies (Cronbach’s α = 0.82–0.90).

#### 2.2.5. Compulsive Internet Use Scale (CIUS)

PUI was assessed using the Compulsive Internet Use Scale (CIUS) [41], a self-report questionnaire measuring problematic patterns of internet use. The instrument consists of 14 items rated on a 5-point Likert scale and is based on DSM-IV criteria for addictive and compulsive behaviors. The CIUS assesses core dimensions of PUI, including loss of control, preoccupation, withdrawal symptoms, interpersonal and intrapersonal conflicts, and coping or mood modification. Previous studies demonstrated good validity as well as high reliability and internal consistency (Cronbach’s α = 0.87–0.90). Total scores range from 0 to 56, with higher scores indicating more PUI [41].

#### 2.2.6. Screen Time

Screen time was operationalized as self-reported private internet use. Participants were asked: “How many hours did you use the internet for private purposes on a weekday?” (in German: “Wie viele Stunden hast Du an einem Wochentag privat das Internet benutzt?”) and “How many hours did you use the internet for private purposes on a weekend day (Saturday/Sunday)?” (in German: “Wie viele Stunden hast Du an einem Tag (Samstag/Sonntag) am Wochenende privat das Internet benutzt?”). Responses were provided in hours per day, with values below one hour coded as 0 according to the original questionnaire instructions. The measure reflects typical daily private internet use rather than use during a specified retrospective period. Separate variables were created for weekday screen time (WD-ST) and weekend screen time (WE-ST). Although the dataset contained additional questions regarding specific internet applications, these variables were not included in the present analyses as the aim was to examine overall private internet use rather than application-specific patterns of use.

### 2.3. Data Analysis

Specific psychiatric diagnoses were not recorded for the present analyses, as the focus was placed on transdiagnostic factors rather than disorder-specific differences. To examine the influence of intrapersonal factors (i.e., self-esteem, emotion regulation strategies), emotional and behavioral problems (internalizing and externalizing symptoms), FoMO, and PUI on screen time, multivariate linear regression analyses were conducted separately for WD-ST and WE-ST. This distinction was made because WD-ST and WE-ST differed significantly, as indicated by prior paired-samples *t*-tests.

Prior to the regression analyses, Pearson correlation analyses were conducted to examine bivariate associations between all predictors and the two screen time outcomes (WD-ST and WE-ST). *p*-values were adjusted for multiple comparisons using the Benjamini–Hochberg False Discovery Rate correction (FDR-BH) [42]. All associations originally reported as *p* < 0.001 were conservatively treated as *p* = 0.001 for the purpose of this correction.

Separate models were estimated because weekday and weekend screen time differed significantly in the sample, suggesting that predictors may show different patterns of association across these contexts. These analyses were exploratory and were not intended to formally test differences in predictor effects between weekdays and weekends. Follow-up models included predictors that were statistically significant in the initial regression analyses, as well as theoretically relevant variables that showed significant bivariate associations with the outcome and/or trend-level associations in the multivariable model. In the first step, PUI was entered as the primary predictor. In the second step, the remaining selected predictors were entered simultaneously. Changes in explained variance (ΔR^2^) and Bayesian Information Criterion (BIC) values were used to evaluate model improvement.

Due to missing data across study variables, sample sizes varied between analyses. Missing values ranged from 10 to 28 cases across variables. Listwise deletion was applied for all statistical analyses.

To evaluate the potential influence of between-study heterogeneity resulting from the pooled dataset, additional sensitivity analyses were conducted including study source as a categorical factor in the regression models [43]. The inclusion of study source did not materially alter the pattern of results.

As the present study is based on pooled secondary data, sample size was determined by data availability rather than prospective planning. To evaluate the adequacy of the obtained sample, a sensitivity power analysis [44] was conducted for the overall regression model. The significance level was set at α = 0.05, and the desired statistical power was 0.80. The overall regression models included seven substantive predictors and two covariates (age and sex), resulting in nine predictors in total. For the WD-ST regression model, the final sample comprised 123 participants. The analysis indicated a minimum detectable R^2^ of 0.119, corresponding to an explained variance of approximately 12.0%, and a critical value of F = 1.96 for the overall model to be considered statistically significant. For the WE-ST regression model the final sample comprised 124 participants. The analysis indicated a minimum detectable R^2^ of 0.120 (12.0% explained variance) for the overall model to be considered statistically significant.

Prior to analyses, assumptions of linear regression were examined, including normality of residuals, homoscedasticity, multicollinearity, and influential observations. Visual inspection of Q–Q plots indicated minor deviations from normality, with slight right-skewness in the residual distribution. However, residuals were overall considered sufficiently normally distributed for linear regression analyses. Multicollinearity diagnostics indicated no evidence of problematic collinearity among predictors (VIFs = 1.14–2.26; tolerance values = 0.442–0.880). Multivariate outliers were assessed using Mahalanobis distance and Cook’s distance diagnostics [43]. Additional sensitivity analyses excluding influential observations yielded comparable results, indicating that the findings were robust.

All statistical analyses were conducted using R (version 4.4.1) [45] and Jamovi [46] with a significance level of *p* < 0.05.

## 3. Results

### 3.1. Descriptives

The sample consisted of 173 adolescents (115 female, 58 male) aged between 12 and 18 years. Female participants had a mean age of 15.4 years (SD = 1.44) and male participants a mean age of 15.6 years (SD = 1.52). Average WD-ST was 5.16 h (SD = 3.32) among female and 4.91 h (SD = 3.16) among male participants. WE-ST was higher, with female participants reporting 6.58 h (SD = 3.73) and male participants 6.94 h (SD = 4.18). WD-ST and WE-ST for the whole sample were compared using a paired-samples *t*-test. Adolescents reported significantly higher WE-ST (M = 6.70 h, SD = 3.88) than WD-ST (M = 5.08 h, SD = 3.26, t(153) = −7.45, *p* < 0.001, dz= 0.61, indicating a difference of moderate effect size. Descriptive statistics for all study variables are presented in Table 2.

**Figure 1:**
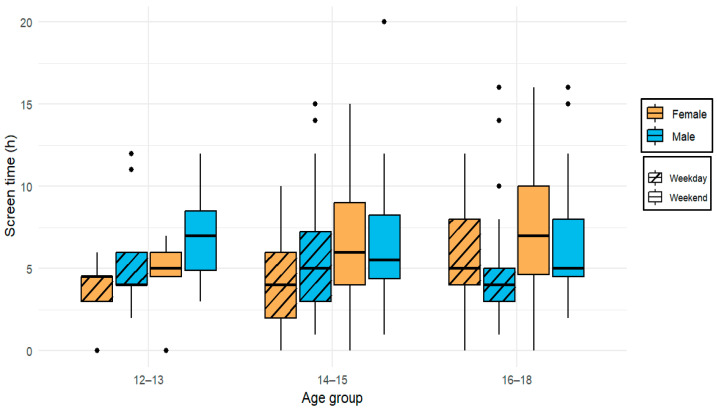
Screen time (hours) by age group, gender, and day type (weekday vs. weekend). *Note*: Distribution of daily screen time (hours) across age groups (12–13, 14–15, and 16–18 years), stratified by gender (female, male) and day type (weekday and weekend). Boxplots display the median, interquartile range (IQR), whiskers extending to 1.5 times the IQR, and individual outliers. Screen time was generally higher on weekends than on weekdays across all age and gender groups. Within each age group, female and male distributions are displayed side by side to facilitate comparison.

Mean WD-ST and WE-ST showed similar overall patterns. When separated by sex and day type, mean WE-ST in the female group increased with age, mean WE-ST in the male group was slightly higher in the 12–13-year age group and decreased across older age groups (see Figure 1).

Pearson correlation analyses were conducted to examine associations between all predictors (self-esteem, adaptive and maladaptive emotion regulation strategies, internalizing and externalizing symptoms, FoMO, and PUI) and screen time outcomes. *p*-values were adjusted for multiple comparisons using the Benjamini–Hochberg FDR correction [42]. WD-ST was significantly associated with externalizing symptoms (r = 0.33, *p* = 0.002), FoMO (r = 0.20, *p* = 0.017), and PUI (r = 0.36, *p* = 0.002). WE-ST showed significant associations with externalizing symptoms (r = 0.23, *p* = 0.017) and PUI (r = 0.37, *p* = 0.002). Detailed correlation coefficients are presented in Table A1 in the Appendix A.

### 3.2. Predictors of Weekday Screen Time (WD-ST)

A multiple linear regression analysis was conducted to examine predictors of WD-ST. The overall regression model was significant, F(9,113) = 3.93, *p* = 0.002, explaining 23.8% of the variance in WD-ST (R^2^ = 0.238).

PUI significantly predicted higher WD-ST, B = 0.089, SE = 0.027, β = 0.310, t = 3.31, *p* = 0.002. In addition, externalizing symptoms were significantly associated with higher WD-ST, B = 0.074, SE = 0.030, β = 0.235, *p* = 0.024. FoMO was not significantly associated with WD-ST, B = 0.049, SE = 0.025, β = 0.170, *p* = 0.072.

Age, self-esteem, adaptive and maladaptive emotion regulation strategies, internalizing symptoms, and sex were not significantly associated with WD-ST (all ps > 0.05).

#### Follow-Up WD-ST

In the first step, PUI was entered and explained 15.0% of the variance in WD-ST (R^2^ = 0.150, BIC = 662). In the second step, externalizing symptoms and FoMO were added, resulting in an increase in explained variance to 21.7% (R^2^ = 0.217, ΔR^2^ = 0.067, BIC = 661).

In the final model, PUI emerged as the strongest predictor of WD-ST, B = 0.083, SE = 0.026, β = 0.277, t = 3.18, *p* = 0.006. In addition, higher levels of externalizing symptoms were significantly associated with increased WD-ST, B = 0.070, SE = 0.028, β = 0.219, t = 2.53, *p* = 0.021. FoMO was not significantly associated with WD-ST, B = 0.047, SE = 0.024, β = 0.156, t = 1.94, *p* = 0.072. Complete values are displayed in the Appendix A (see Table A2).

Although Model 2 explained more variance than Model 1 (ΔR^2^ = 0.067), the change in BIC was negligible (ΔBIC = 1), suggesting no meaningful difference in model fit.

### 3.3. Weekend Screen Time (WE-ST)

A multiple linear regression analysis was conducted to examine predictors of WE-ST. The overall regression model was significant, F(9,114) = 4.15, *p* = 0.002, explaining 24.7% of the variance in WE-ST (R^2^ = 0.247).

PUI significantly predicted higher WE-ST, B = 0.117, SE = 0.032, β = 0.344, t = 3.70, *p* = 0.002. FoMO was not significantly associated with WE-ST, B = 0.057, SE = 0.030, β = 0.166, *p* = 0.073.

Sex was not significantly associated with weekend screen time (*p* = 0.093). Age, self-esteem, adaptive and maladaptive emotion regulation strategies, and internalizing symptoms were not significantly associated with WE-ST (all ps > 0.05).

#### Follow-Up WE-ST

In the first step, PUI was entered as a predictor and significantly explained 11.3% of the variance in WE-ST, R^2^ = 0.113, F(1,145) = 18.56, *p* = 0.002, BIC = 821.

In the second step, FoMO and sex were added to the model, resulting in a small increase in explained variance to 12.7% (ΔR^2^ = 0.014). However, this increase was not statistically significant, F(2,143) = 1.13, *p* = 0.341. The final model remained significant overall, R^2^ = 0.127, F(3,143) = 6.95, *p* = 0.002, but showed a higher BIC value (BIC = 829), suggesting no substantial improvement in model fit compared to the simpler model.

In the final model, PUI remained a significant predictor of higher WE-ST, B = 0.121, SE = 0.029, β = 0.328, t = 4.14, *p* = 0.002. Neither FoMO, B = 0.029, SE = 0.031, β = 0.078, t = 0.95, *p* = 0.344, nor sex, B = 0.936, SE = 0.679, β = 0.234, t = 1.38, *p* = 0.185, significantly predicted WE-ST. Complete values are displayed in the Appendix A (see Table A3).

## 4. Discussion

### 4.1. PUI as the Strongest Predictor of Screen Time Across Weekdays and Weekends

The present findings consistently identified PUI as the strongest predictor of screen time in our clinical CAP sample. In the follow-up hierarchical regression analyses, PUI accounted for 15.0% of the variance in WD-ST when entered as the sole predictor in the first model step and remained the strongest and only significant predictor in the final WE-ST follow-up model. This pattern is consistent with the I-PACE model’s conceptualization of PUI as emerging from the interplay of individual vulnerability factors, including maladaptive coping and impaired self-regulation, with affective-cognitive responses to specific online stimuli [16,17]. Within this framework, elevated screen time is not merely a consequence of higher PUI severity but reflects a shared underlying regulatory deficit, whereby reduced executive functions may operate as both a vulnerability factor and a consequence of PUI progression. Consistent with more recent interpretations of the model, this deficit is likely to manifest across both gratification-seeking and compensatory internet use, with compensation becoming increasingly dominant at later stages of PUI development [17]. The association between PUI and screen time should be interpreted with some caution, as the CIUS includes items assessing preoccupation with internet use, difficulties controlling online behavior, and time spent online. Consequently, some degree of conceptual overlap between the predictor and outcome measure may have contributed to the observed association.

The screen time values observed in the clinical sample, averaging 5.08 h on weekdays and 6.70 h on weekends, exceed the maximum of two hours of daily recreational screen time recommended by the most permissive international guidelines for children over five years and adolescents [47], and are markedly higher than values reported in population-based adolescent samples. Even the heaviest-use trajectory group in a Swiss longitudinal cohort followed from age 11 to 17 averaged approximately three hours of daily screen time across videogaming and internet use [48]. In a large pre-adolescent sample, two or more hours of daily screen time was associated with clinically relevant internalizing disorder diagnoses as assessed via structured clinical interview [49]. These screen time values are further contextualized by PUI rates reported in inpatient psychiatric settings, where prevalence considerably exceeds community estimates, reaching 34% full PUI and 62% subthreshold PUI [5,6].

Taken together, these findings suggest that PUI remained the strongest predictor of screen time after adjustment for self-esteem, emotion regulation, and internalizing symptoms. This implies that quantitative digital engagement in clinical samples cannot be reduced to duration alone but reflects its compulsive character. Consistent with evidence that school-based screen time reduction interventions yield only modest effects even under controlled conditions [50], routine restriction of screen time without addressing underlying PUI may therefore have limited therapeutic utility. This is consistent with evidence indicating that the type, content, and context of internet use matter more than duration alone [14], as demonstrated in a nationally representative British adolescent sample showing that social media and internet use were more strongly associated with compromised mental health than television viewing or gaming [51].

### 4.2. Externalizing Symptoms and FoMO as Exploratory Associations with Weekday Screen Time

Externalizing symptoms emerged as a significant predictor of WD-ST (β = 0.219, *p* = 0.021), whereas FoMO showed a consistent but non-significant positive association with WD-ST across both the full (β = 0.170, *p* = 0.072) and follow-up regression models (β = 0.156, *p* = 0.072). Neither variable was significantly associated with WE-ST. Given the theoretical relevance of FoMO in clinical adolescent populations, this non-significant but directionally consistent pattern is discussed below as a potentially meaningful signal warranting further investigation.

This pattern is consistent with longitudinal community-based evidence showing that externalizing symptoms predict higher subsequent weekday screen time, with the strongest effect observed for rule-breaking behavior [22]. Although the present findings do not permit formal conclusion regarding differences between weekday and weekend effects, the pattern may be consistent with a moderating role of daily structure imposed by school or vocational obligations, which constrain available screen time on weekdays and may amplify the visibility of symptom-driven digital engagement within these bounded time windows. Externalizing behavior problems, including impulsive, oppositional, and dysregulated conduct, may interfere with compliance with these structured demands and drive compensatory digital engagement during or after structured time periods. The non-significant but consistent association of FoMO with weekday screen time may similarly reflect heightened awareness of peer social activities during periods of reduced personal freedom, though this interpretation remains speculative.

FoMO has been conceptualized as rooted in unmet belonging needs, a developmental dynamic particularly salient during adolescence when peer belonging and social comparison are especially prominent [34]. The observed pattern, whereby FoMO showed a non-significant but consistently positive association with WD-ST while no comparable pattern was observed for WE-ST, may reflect the perceived cost of being offline during school days, when adolescents are physically separated from their peer networks. At weekends, when structured obligations are reduced and social access less constrained, the perceived cost of being offline may diminish accordingly. Although the association did not reach statistical significance, this pattern may tentatively indicate that FoMO’s relationship with screen use may be context-dependent rather than stable across contexts. However, this interpretation should be considered exploratory and requires confirmation in future studies, particularly in clinical adolescent populations in which peer belonging is already compromised [52]. These dynamics are consistent with broader developmental frameworks emphasizing the relational and neurobiological foundations of socioemotional development during adolescence, whereby early caregiving experiences shape the self-regulatory and affiliative capacities that modulate vulnerability to peer-driven digital engagement [28]. This interpretation should nonetheless be treated with caution given the non-significant *p*-values.

The absence of significant associations for externalizing symptoms and FoMO in the weekend model may indicate potentially different patterns of associations across contexts, although the present analyses did not directly test whether these effects differ significantly between weekdays and weekends. If replicated, these findings may have implications for screen time guidelines that address total weekly duration without distinguishing between structured and unstructured time periods [12,14]. The present findings suggest a potential context-dependent pattern that may provide clinically informative insights beyond aggregate weekly estimates, although this possibility requires further investigation.

### 4.3. Non-Significant Predictors: Self-Esteem, Emotion Regulation, and Internalizing Symptoms

Self-esteem, emotion regulation strategies, and internalizing symptoms were not significantly associated with screen time in either model. In a clinical sample with uniformly elevated psychopathological burden, range restriction likely attenuated these associations. The relationship between emotion regulation and digital behavior in high-PUI samples may operate primarily through PUI rather than as a direct pathway. Using the same FEEL-KJ instrument, maladaptive emotion regulation emerged as the sole significant predictor of PUI remission over one year [53]. At higher PUI levels, internet use itself progressively assumes a regulatory function, weakening the direct association between temperament-based regulatory difficulties and emotion regulation strategies, in line with the I-PACE model [16,17,54]. Self-esteem, in contrast, appears to function more as a moderator than as a direct predictor, an effect that would not be captured by the present design [26]. The inclusion of self-esteem was theoretically motivated by its documented role as a moderator of social media effects on depressive symptoms in young adult samples [26] and its negative association with social networking site use across adolescent and young adult populations, particularly in the context of problematic use [30]. Its null finding in the present study is therefore interpreted not as evidence against a relationship, but as reflecting the limits of a direct-effects model in a sample where psychopathological burden is uniformly elevated, and range restriction is likely.

Although the introduction highlighted gender-differentiated patterns of internet use, with boys predominantly engaging in gaming and girls in social media and social communication, sex was included as a covariate rather than a moderator in the present analyses. The present sample was not sufficiently powered to support gender-stratified regression models, and collapsing across gender may have obscured differential associations. Whether the observed associations between PUI, externalizing symptoms, and screen time differ in magnitude or direction across gender groups remains an open question that warrants investigation in larger clinical samples. It should be noted that the individual-level predictors examined here do not operate in isolation, and that family context, particularly parental digital media use and the quality of parent–child communication, likely shapes the regulatory capacities and digital engagement patterns observed in this sample [20].

### 4.4. Strengths, Limitations, and Clinical Implications

The present study has several methodological strengths. The sample comprised 173 adolescents (66.5% female, mean age = 15.4 years for girls and 15.6 years for boys), reflecting the clinical heterogeneity typical of inpatient and day clinic settings. It employs a clinical sample of CAP inpatients and day clinic patients, a population that is systematically underrepresented in the screen time literature despite its clinical relevance [5,6]. The simultaneous examination of WD-ST and WE-ST as distinct outcomes allowed for the identification of context-specific predictor patterns that a composite measure would have obscured. Regression assumptions were systematically verified, and sensitivity analyses excluding influential observations yielded robust results. The transdiagnostic focus of the present analyses represents an additional methodological strength. Rather than focusing on disorder-specific effects, the study examined psychological and behavioral factors that may operate across diagnostic boundaries. This approach is consistent with dimensional frameworks of psychopathology and may be particularly appropriate for screen time research, where associations with mental health often extend across traditional diagnostic categories.

Several limitations warrant consideration. First, the cross-sectional design precludes causal inference. Although PUI emerged as a significant predictor of screen time, the reverse relationship remains equally plausible, with greater screen exposure contributing to the development or maintenance of problematic internet use over time. Second, screen time was assessed via self-report, which may underestimate actual usage in adolescent populations [14] and could have attenuated observed associations.

A further limitation concerns the temporal reference of the measures. Both screen time and PUI were assessed retrospectively, reflecting digital engagement patterns prior to clinical admission rather than during the inpatient or day clinic stay. Consequently, the findings cannot be interpreted as reflecting digital engagement within the treatment setting, nor can conclusions be drawn regarding the stability of the observed associations across treatment effects.

The absence of a non-clinical comparison group limits the generalizability of findings, as it remains unclear whether the observed predictor patterns represent clinical-specific phenomena or amplified versions of associations present in the general population. The sample was drawn from a single hospital location in Salzburg, Austria, and findings may not generalize to clinical populations in other countries, healthcare systems, or cultural contexts where patterns of digital media use, clinical care, and family structure may differ.

An additional restriction concerns the absence of diagnosis-specific analyses. The present study adopted a transdiagnostic approach and therefore focused on psychological processes that may operate across diagnostic categories rather than on disorder-specific effects. Consequently, it remains unclear whether the observed associations are equally applicable across different clinical presentations or are driven primarily by particular diagnostic subgroups. Future research should investigate whether these relationships vary across diagnostic categories while maintaining a transdiagnostic perspective on underlying mechanisms.

The present study did not assess parental internet use or the quality of parent–child communication around digital media. Both factors have been identified as among the strongest correlates of adolescent PUI [20] and may account for variance in screen use beyond the individual-level factors examined here. Consequently, the observed associations between PUI, externalizing symptoms, and screen time should be interpreted within the broader family context, which was not captured by the present design.

In addition, platform-specific analyses were not conducted, potentially obscuring differential effects of gaming-dominated versus social media-dominated usage profiles, which have been shown to produce opposing associations with PUI outcomes in adolescent samples [6,19].

Another limitation concerns the timing of data collection. The pooled dataset included studies conducted between 2021 and 2024, with a substantial proportion of participants recruited during or shortly after the COVID-19 pandemic. Given evidence of increased screen use among adolescents during this period [55], the reported levels of screen time may not fully reflect post-pandemic patterns of digital engagement. Although sensitivity analyses controlling for study source did not materially alter the findings, the potential influence of pandemic-related behavioral changes should be considered when interpreting the results.

The present findings have several implications for clinical practice in CAP settings. The consistent predictive strength of PUI across both models supports its prioritization in routine clinical screening, using validated instruments such as the CIUS, rather than relying on aggregate screen time duration alone. Such approaches targeting the regulatory deficits underlying PUI are therefore more likely to yield clinically meaningful outcomes than duration-focused restrictions alone [17,56]. For adolescents with elevated externalizing symptoms, providing structured alternatives for peer connection during weekday periods may additionally reduce compensatory digital engagement. Given that parental internet use is among the strongest correlates of adolescent PUI [20], family-based components addressing digital media use warrant integration into discharge planning and outpatient follow-up.

## 5. Conclusions

The present study indicates that PUI and externalizing symptoms were the variables most consistently associated with screen time in a clinical CAP sample. Externalizing symptoms were significantly associated with WD-ST but not WE-ST, while FoMO showed a positive but non-significant association with WD-ST only. These findings suggest that screen time duration alone may not fully capture clinically relevant aspects of digital engagement and support consideration of PUI-focused assessment as an additional indicator in clinical contexts [14]. Although the observed differences between weekday and weekend screen time may point to context-dependent patterns of association, the present cross-sectional findings do not allow conclusions regarding distinct underlying mechanisms. Similarly, while these findings are compatible with theoretical perspectives emphasizing the influence of daily structure on problematic digital behavior [16,17], they should be interpreted as exploratory and require further empirical validation.

These observations may nevertheless have implications for clinical monitoring and for considering the context in which digital media assessments are conducted. The present findings may also inform ongoing policy discussions regarding screen time regulation in young people. Recent clinical guidelines, including those issued by the Italian Society of Pediatrics [2], reflect growing pediatric concern that excessive and unregulated digital media exposure may be associated with adverse health outcomes and support structured preventive approaches. These recommendations, developed primarily from community-based evidence, remain important at the population level. However, the present findings suggest that focusing exclusively on reducing screen time duration may not sufficiently address the broader psychological and behavioral processes associated with digital engagement in clinical populations. Consistent with previous intervention research showing modest effects of screen time reduction alone and inconsistent effects on physical health outcomes [50], the current results support further investigation into whether interventions addressing PUI-related processes may complement duration-focused approaches.

## Figures and Tables

**Table 1:** Characteristics of the contributing studies and their contribution to the regression analysis.

Study (Period of Data Collection)	N	Age (M, SD)	WD-ST (M, SD)	WE-ST (M, SD)	Intervention	Setting	Complete Cases (N, %)
1. Dance (2023 to 2024)	N = 19 (female = 12) (1 exclusion in the original dataset because age < 12 years)	15.6 (1.30)	5.76 (3.19)	7.41 (4.72)	Art-based (dance)	In-patient, Day clinic	17 (89.47)
2. Photography (2021 to 2022)	N = 64 (female = 33) (3 exclusions of original dataset because age > 18 years in the merged data set)	15.4 (1.78)	4.72 (3.23)	6.46 (3.79)	Art-based (portrait photography)	In- and Out-patient, Day Clinic	58 (90.62)
3. Choir and other art forms (2021 to 2022)	N = 39 (female = 22)	15.4 (1.25)	5.00 (3.13)	7.21 (4.42)	Art-based (several art forms)	In-patient, Day Clinic	39 (100.0)
4. DBT Skills (2024)	N = 51 (female = 49) (1 exclusion in the original dataset because age < 12 years)	15.4 (1.22)	5.34 (3.47)	6.31 (3.12)	Skill-based training	Out-patient	15 (28.30)

*Note*: Abbreviations: “Dance” refers to study 1 “Effects of dance art on self-esteem and body perception in children and adolescents: A pilot study”, “Photography” refers to study 2 “Me and myself–Self-esteem, self-image, and portrait photography in children and adolescents: A pilot study”, “Choir and other art forms” refers to study 3 “Psychological and physiological effects of choir singing on mentally ill, socially underprivileged, and privileged healthy children and adolescents: An open-label, single-arm controlled study” and DBT-Skills refers to study 4 “Retrospective quality evaluation of an outpatient DBT skills group”. N = sample size; M = mean; SD = Standard deviation; WD-ST = Weekday Screentime; WE-ST = Weekend Screentime. “Complete cases” refer to participants with complete data on the variables included in a given regression model, expressed as a percentage of the study-specific sample size. As predictor availability differed across studies and analyses, the number of complete cases varied between models.

**Table 2:** Descriptive Statistics of Variables Included in the Regression Models.

Variable	N	Missing	Mean	SD	Min	Max
Self-esteem (SEKJ)	152	21	24	9.40	10	48
Adaptive Strategies (FEEL-KJ)	152	21	114	27.6	51	169
Maladaptive Strategies (FEEL-KJ)	152	21	98.5	19.6	11	130
Internalizing Symptoms (YSR)	145	28	28.8	12.0	0	54
Externalizing Symptoms (YSR)	145	28	18.0	10.3	1	45
FoMO (FoMO Scale)	158	15	25.6	11.0	0	50
PUI (CIUS)	163	10	24.3	11.2	0	56
WD-ST hours (subjective report)	154	19	5.08	3.26	0	16
WE-ST hours (subjective report)	155	18	6.70	3.88	0	20

Note: SEKJ = Self-esteem Inventory for Children and Adolescents; FEEL-KJ = Questionnaire for the Evaluation of Emotional Regulation in Children and Adolescents; YSR = Youth Self-Report; FoMO = Fear of Missing Out; CIUS = Compulsive Internet Use Scale; PUI = Problematic Use of the Internet; WD-ST = Weekday Screen Time; WE-ST = Weekend Screen Time; SD = Standard Deviation.

## Data Availability

The data presented in this study are available on request from the corresponding author due to legal reasons in clinical populations.

## Abbreviations

The following abbreviations are used in this manuscript:

CAP	Child and Adolescent Psychiatry
CIUS	Compulsive Internet Use Scale
FEEL-KJ	Fragebogen zur Erhebung der Emotionsregulation bei Kindern und Jugendlichen
FDR	False Discovery Rate
FoMO	Fear of Missing Out
I-PACE	Interaction of Person–Affect–Cognition–Execution
PUI	Problematic Use of the Internet
SD	Standard Deviation
SEKJ	Selbstwertinventar für Kinder und Jugendliche
WD-ST	Weekday screen time
WE-ST	Weekend screen time
YSR	Youth Self-Report

## Appendix A

**Table A1:** Pearson Correlations Between Study Variables.

Variable	1	2	3	4	5	6	7	8	9	10
**1. Age**	-									
**2. Sex**	0.09	-								
**3. Self-esteem**	−0.1	0.29 ***	-							
**4. Adaptive strategies**	−0.03	0.04	0.28 ***	-						
**5. Maladaptive strategies**	0.02	−0.22 **	−0.48 ***	−0.08	-					
**6. Internalizing symptoms**	−0.10	−0.26 **	−0.67 ***	−0.26 **	0.58 ***	-				
**7. Externalizing symptoms**	−0.26 **	−0.15	−0.24 **	−0.11	0.26 **	0.36 **	-			
**8. FoMO**	0.07	−0.28 ***	−0.22 **	0.03	0.27 ***	0.19	0.11	-		
**9. PUI**	−0.11	−0.06	−0.22 **	−0.16	0.16	0.25 **	0.32 ***	0.17	-	
**10. WD-ST**	−0.01	−0.04	−0.06	0.06	0.02	0.13	0.33 ***	0.20 *	0.36 ***	-
**11. WE-ST**	0.05	0.04	−0.08	0.06	−0.11	0.11	0.23 **	0.12	0.37 ***	0.73 ***

Note. Sex was coded female = 0, male = 1. FoMO = fear of missing out; PUI = problematic use of the internet; WD-ST = weekday screen time; WE-ST = weekend screen time. Significance-levels were adjusted using Benjamini-Hochberg-correction (pBH) controlling for False Discovery Rate (FDR = 0.05). pBH < 0.05 *, pBH < 0.01 **, pBH < 0.001 ***.

**Table A2:** Final hierarchical regression model predicting weekday screen time.

			95% CI					95% CI	
Predictor	B	SE	Lower	Upper	t	pBH	β	Lower	Upper
Intercept	0.4069	0.8664	−1.3078	2.1218	0.470	0.639			
PUI	0.0834	0.0262	0.0314	0.1353	3.180	**0.006**	0.277	0.1046	0.450
Ext Symp	0.0704	0.0278	0.0152	0.1255	2.527	**0.021**	0.219	0.0474	0.390
FoMO	0.0470	0.0243	−0.0010	0.0950	1.935	0.072	0.156	−0.0035	0.315

Note: B = unstandardized regression coefficient; SE = standard error; β = standardized regression coefficient; CI = confidence interval. PUI = problematic use of the internet; Ext Symp = Externalizing Symptoms; FoMO = Fear of Missing Out. Significant levels were adjusted using Benjamini-Hochberg-correction (shown as pBH) to avoid False Discovery Rate (FDR = 0.05). Significant effects (pBH < 0.05) are shown in bold.

**Table A3:** Final hierarchical regression model predicting weekend screen time.

			95% CI					95% CI	
Predictor	B	SE	Lower	Upper	t	pBH	β	Lower	Upper
Intercept	2.6272	1.1028	0.4474	4.8071	2.382	0.019			
PUI	0.1211	0.0293	0.0633	0.1790	4.138	**0.002**	0.3277	0.1712	0.484
FoMO	0.0289	0.0305	−0.0313	0.0892	0.949	0.344	0.0782	−0.0846	0.241
Sex:									
Male–Female	0.9364	0.6787	−0.4052	2.2779	1.380	0.185	0.2337	−0.1012	0.569

Note: B = unstandardized regression coefficient; SE = standard error; β = standardized regression coefficient; CI = confidence interval. Female served as the reference category for sex. PUI = Problematic Use of the Internet; FoMO = Fear of Missing Out. Significant levels were adjusted using Benjamini-Hochberg-correction (shown as pBH) to avoid False Discovery Rate (FDR = 0.05). Significant effects (pBH < 0.05) are shown in bold.
